# Propagation of errors in citation networks: a study involving the entire citation network of a widely cited paper published in, and later retracted from, the journal Nature

**DOI:** 10.1186/s41073-016-0008-5

**Published:** 2016-05-03

**Authors:** Paul E. van der Vet, Harm Nijveen

**Affiliations:** 1 Human Media Interaction Group, Department of Computer Science, University of Twente, Drienerlolaan 5, Enschede, 7522 NB the Netherlands; 2 ZGT Academy, Ziekenhuisgroep Twente, Zilvermeeuw 1, Almelo, 7609 PP the Netherlands; 3 Bioinformatics Laboratory, Wageningen University, Droevendaalsesteeg 1, Wageningen, 6708 PB the Netherlands; 4 Wageningen Seed Lab, Laboratory of Plant Physiology, Wageningen University, Droevendaalsesteeg 1, Wageningen, 6708 PB the Netherlands

**Keywords:** Retraction, Citing behaviour, Citation network

## Abstract

**Background:**

In about one in 10,000 cases, a published article is retracted. This very often means that the results it reports are flawed. Several authors have voiced concerns about the presence of retracted research in the memory of science. In particular, a retracted result is propagated by citing it. In the published literature, many instances are given of retracted articles that are cited both before and after their retraction. Even worse is the possibility that these articles in turn are cited in such a way that the retracted result is propagated further.

**Methods:**

We have conducted a case study to find out how a retracted article is cited and whether retracted results are propagated through indirect citations. We have constructed the entire citation network for this case.

**Results:**

We show that directly citing articles is an important source of propagation of retracted research results. In contrast, in our case study, indirect citations do not contribute to the propagation of the retracted result.

**Conclusions:**

While admitting the limitations of a study involving a single case, we think there are reasons for the non-contribution of indirect citations that hold beyond our case study.

**Electronic supplementary material:**

The online version of this article (doi:10.1186/s41073-016-0008-5) contains supplementary material, which is available to authorized users.

## Background

Scientific investigation is difficult and fallible, and its practitioners are only human. Results believed to be firm may turn out to be not reproducible or outright wrong or even faked. If detected, this leads to retraction of an article. Retraction is a dramatic event. It damages careers and may incur large costs [1, 2]. How publishers are to handle retractions is currently debated [3].

Retraction of a published article is a rare event, but its incidence is on the rise from roughly one in 100,000 cases before the year 2000 to one in 10,000 cases in the last decade [4]. The reasons for retraction vary and can be classified roughly into two categories: scientific misconduct on the one hand, and error or lack of reproducibility on the other. Earlier research found error to be the main cause for retraction [5]. Later studies find that misconduct is the main cause [6–8], although Couzin and co-workers point out that even outright fraud not always leads to retraction [9]. Behaviour of both authors and institutions is said to account for the rise of misconduct among retractions [10]. A few repeat offenders heavily bias retraction rates [4]. Repeat offenders are said to be responsible for roughly half of all retraction cases [4].

Although the retraction of a paper is normally interpreted as signalling that the results of the paper are flawed, this is not guaranteed. In our case study, see below, the matter appears far from settled. Even fraud may turn up results that are later found to be correct. We will therefore not speak about “flawed” or “erroneous” results but rather about “retracted” results.

An important question is what damage is done by the retracted article. A retracted result is formally no longer part of the body of science. Therefore, retractions must be advertised to prevent spreading of retracted results. The blog *Retraction Watch* is providing an invaluable service in this respect. A review of the literature on retractions has recently been published by the initiators of the blog, Marcus and Oransky [11]. Retracted results pollute their citation environments [11, 12]. Examples of a retracted result still cited years after its retraction have been reported [13–15]. A number of studies report on how often retracted articles are cited both before and after retraction [16–18]. Where one report finds that citation rates drop by approximately 35 % after retraction [16], another report finds no significant decrease in citation rates after retraction [17]. Retracted and non-retracted articles alike are all subject to attention decay with the result that most are eventually largely forgotten [19].

Particularly in the medical literature, there is the danger that patients are put at risk by what is concluded in articles that later have to be retracted. The Wakefield case is probably the most famous example. Wakefield and co-workers claimed to have found an association between measles vaccine and autism [20] but their article was retracted because of fraud 12 years later [21]. The false association has lingered on since then and may have caused unnecessary deaths through parents refusing measles vaccination of their children [22]. Treatments based on retracted articles put patients at risk [23]. Neale and co-authors find no such cases in their study involving 102 articles retracted because of misconduct [17], while Begley and co-authors conclude the opposite [24] and Couzin and co-workers provide a concrete example [9].

Chen and co-workers point to the following scenario [25]. Suppose an article *A* is retracted and that *A* has been cited in a positive way by *B*, *C* and *D*. In the worst case, *A*’s retracted findings support conclusions drawn in these papers. *B*, *C* and *D*, in turn, are cited by yet other papers. *A*’s retracted results may again be essential ingredients of the argument of these other papers. Because *A*’s conclusions are retracted, the conclusions in all these papers should be re-examined. Chen and co-workers have conducted a large-scale investigation that precluded them from inspecting individual articles [25]. Therefore, they did not find examples of their scenario. Fulton and co-workers, on the other hand, have studied a single case in detail but have concentrated only on articles that directly cite the retracted article [26]. Like that paper, we focus on a single case because that way we have the possibility to study the contents of the papers involved. We study articles that directly cite a retracted article both before and after retraction. Unlike Fulton and co-workers [26], we identify the entire citation environment of the retracted paper. We thus also inspect articles that are connected to the retracted article through a chain of citations in order to find out whether in this case the scenario identified in [25] has become a reality.

## Methods

We have selected a particular paper published in December 2012 because it was published in *Nature* and because it deals with necrosis and with sirtuins (a class of proteins). Briefly, in [27] (called “the Narayan paper” from now on), Narayan and co-workers claim that inhibition of sirtuin-2 blocks cellular necrosis induced by TNF- *α*. The Narayan paper was retracted in February 2014 [28] when a number of groups reported they were unable to reproduce its findings [29]. Meanwhile, the National Institutes of Health, the parent organisation of Narayan and most co-authors, had published an invention based on the Narayan paper as being available for licensing [30]. We have not found a retraction of this notice. When we inspected the list of publications at the personal website of the last senior author (T. Finkel) in September, 2015, the Narayan paper was there but the retraction went unmentioned. What is more, two papers published too late to be included in the present research suggest that the results of Narayan paper are not flawed after all [31, 32]. There is no overlap between the authors of these two papers and the authors of the Narayan paper, nor is there any overlap with the authors of [29], the paper that prompted the retraction.

We need some lightweight formal apparatus to describe our definitions. We base these definitions on the primitive relation Cites(*x*,*y*) with the obvious meaning that document *x* cites document *y*. A *citation chain* is an ordered list *L*=〈*D*_1_,…,*D*_*i*_,*D*_*i*+1_,…*D*_*n*_〉 such that

$$ \forall i \ D_{i} \in L \wedge D_{i+1} \in L \Rightarrow \text{Cites}(D_{i+1}, D_{i}) $$

In other words, every document in the chain (except, for trivial reasons, the last) is cited by the document following it. *D*_2_, the second document in the chain, is a document that *directly* cites the first document in the chain, *D*_1_. All documents further in the citation chain, in other words, all *D*_*i*_ such that *i*>2, will be said to *indirectly* cite *D*_1_, even though these documents do not acknowledge the existence of *D*_1_. In our case, *D*_1_ is always the Narayan paper.

For a given paper *P*, we define the *citing collection* *C* as the set of all papers that either directly or indirectly cite *P*:

$$ C = \{ x \ | \ \text{Cites}(x,P) \ \vee \ \exists y \ y \in C \wedge \text{Cites}(x, y) \} $$

Finally, we define the *citation network* of *P* as the directed graph 〈*N*,*E*〉 with

$$ N = \{ P \} \cup C $$

with *C* the citing collection of *P* as above and

$$ E = \{ \langle x,y \rangle \ | \ x \in N \wedge y \in N \wedge \text{Cites}(x,y) \} $$

In our case, *P* is the Narayan paper.

We inspected citations in two sessions, the first in March, 2014, and the second 1 year later, in March, 2015. In both sessions, we used Elsevier’s search engine for scientific publications Scopus. Starting with the papers that cite the Narayan paper, we followed all citations until we arrived at a paper that at the time was not or not yet cited. We had Scopus produce lists of citing papers in BibTE X format. BibTE X needs a unique identifier. Scopus constructs this identifier by concatenating the name of the first author, the year of publication, and the page number at which the article starts. Thus, the identifier for the Narayan paper becomes “Narayan2012199”. For reasons having to do with limitations of the programmes we used, we had to turn the identifiers allocated by Scopus into a simple ASCII form by removing diacriticals and non-alphanumeric characters. “Martínez-Redondo” becomes “MartinezRedondo”, “Nührenberg” becomes “Nuhrenberg”, and so on. The way in which Scopus constructs its identifiers and our further simplification of the Scopus identifiers may lead to the same identifier pointing to two (or even more) different articles, but in the restricted set used for the present experiment we have not found this. We used the programming language Prolog to process the files with citing papers produced by Scopus. We thus obtained the two complete citation networks, one for 2014, the other for 2015.

We read all articles that directly cite the Narayan paper to find out which text accompanies the citation. In particular, we were interested to learn whether the retraction had been acknowledged. Furthermore, to find out whether the results reported in the Narayan paper had spread to papers that indirectly cite the Narayan paper, we reasoned that any such paper should match keywords such as “sirt”, “sirtuin”, “SIRT2”, “necrosis”, “necrotic”, “necroptosis”, and similar. We found that there is sometimes a time gap between publication of an article and the moment it is incorporated into the Scopus database. Allowing for this latency, in July, 2015, we used Scopus to perform a literature search on articles published after 2011 with the search term sirt* AND necro*, where the asterisk is the Kleene star standing for zero, one or more non-white characters. We then determined the overlap between this set, on the one hand, and the 2014 and 2015 citing collections, on the other. Any article that is a member of the overlap set and furthermore does not contain a direct citation to the Narayan paper is a candidate for inspection on spreading of the retracted result through a citation chain. We read all those papers, too, to find out whether the results of the Narayan paper are mentioned as such and, if so, whether we can trace this back to the Narayan paper by following the citation chain.

## Results and discussion

Briefly, articles that directly cite the Narayan paper just repeat the (retracted) result, with two exceptions. By contrast, in papers that indirectly cite the Narayan paper there is no trace of the retracted result.

### Results

**Fig. 1:**
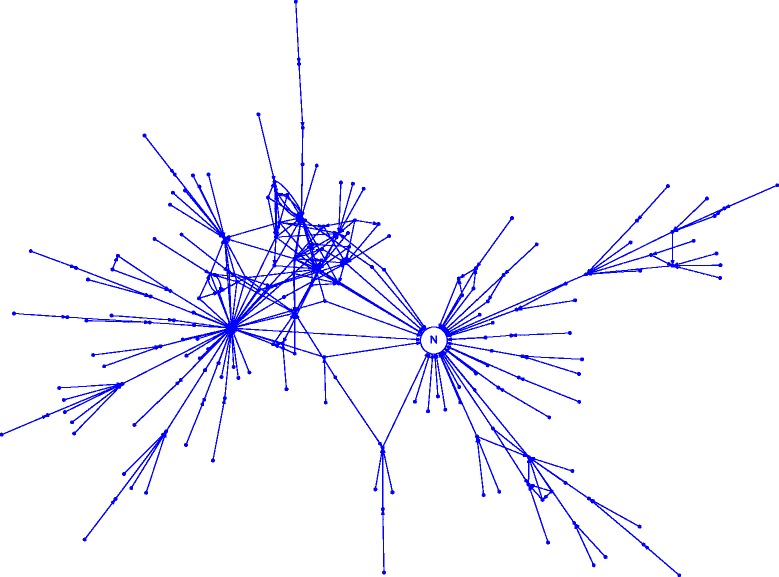
The 2014 citation network for the Narayan paper. Node names have been replaced by dots. Every *node*stands for a paper. Every *arrow*stands for a citation relation. The *arrow*points from the citing paper to the paper that is being cited. The Narayan paper is represented by the *blue circle* with the *N* inside

**Fig. 2:**
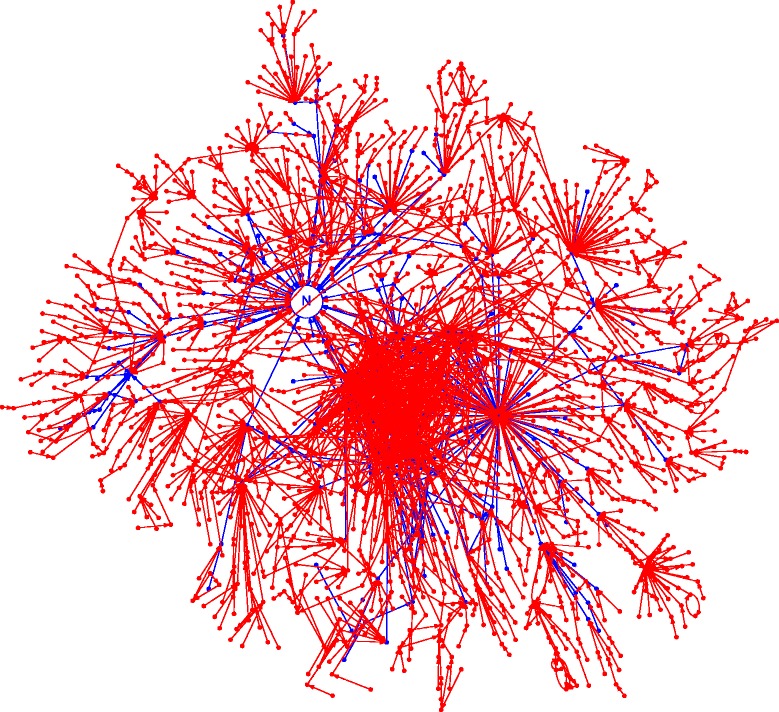
The 2015 citation network of the Narayan paper. The network is shown here as a combination of the 2014 network (*blue*) and the 2015 additions (*red*). As is Fig. 1, every *node*stands for a paper and every *arrow*for a citation relation. The Narayan paper is represented by the *blue circle* with the *N* inside

In the two sessions, we collected two complete citation networks. The networks are not proper trees because citation cycles occur in both. The 2014 network (Fig. 1) is a subgraph of the 2015 network (Fig. 2). The growth is spectacular. See Table 1 for the main counts. The supplementary material contains, for every article that directly cites the Narayan paper, the sentences or passages that contain the citation (Additional file 1). The supplementary material also contains a complete specification of the 2014 and 2015 networks in the form of dot files [33] (Additional files 2 and 3). A visual rendering of the 2014 graph, split over two figures for readability, is provided as scalable PDF files (Additional files 4 and 5). The accompanying BibTE X file (Additional file 6) relates the identifiers in the dot files and in the figures to the complete bibliographic descriptions.

**Table 1:** Summary of counts, see main text for information

	2014	2015
Citation networks		
# articles	187	1626
# citation relations	277	2457
Cited or not		
# articles not (yet) cited	118 (63 %)	1037 (64 %)
# cited articles	69 (37 %)	589 (36 %)
Articles that directly cite the Narayan paper		
# articles that directly cite the Narayan	37	57
paper		
Of which are Reviews	18	28
Of which are Original contributions	17	26
Where:		
# citations in the *Introduction*	12	14
# citations in the *Materials &*	1	1
*Methods* section		
# citations in the *Results*	1	3
# citations in the *Discussion*	9	17
Overlap counts		
# directly citing papers in overlap	7	10
# indirectly citing papers in overlap	1	10

The number of Reviews and the number of Original contributions do not add up to the total number of articles that directly cite the Narayan paper. In 2014, apart from Reviews and Original contributions we have the paper that prompted the retraction [29] and a note; in 2015, we have one further note. Also, the retraction itself is left out of all counts. The numbers of citations in the various sections of Original contributions add up to totals larger than the number of Original contributions because in some Original contributions there are several citations. The overlap counts refer to the overlap of the 2014 and 2015 citing collections, on the one hand, and the July 2015 search result on the search term “sirt* AND necro*” limited to articles published after 2011

The retraction of the Narayan paper is absent in the 2014 network even though 2 weeks had passed between retraction and our Scopus search. Scopus displays some latency. In the two networks, about two thirds of the papers had not or not yet been cited at the time the network was collected. Of the papers that are cited, the median citation count is 1. The distribution of citation frequencies follows a Zipf law, in line with what has been reported in the literature [34]. In our case, only a few papers are cited more than once. The most often cited paper in both networks is the review paper by Kaczmarek c.s. [35] that directly cites the Narayan paper. The Kaczmarek paper had collected 42 citations in 2014 and 111 citations in 2015. The next most often cited paper is the Narayan paper itself.

Of the 37 papers in the 2014 network that directly cite the Narayan paper, one is the paper that prompted the retraction [29], and one paper [36] is in fact a summary of the Narayan paper in the *Reviews and Comments* section of the *Nature* issue in which the Narayan paper was published. In the 2015 network, we have one further directly citing article characterised as a note. For further data, see Table 1. With two exceptions, both from the 2015 network, none of the directly citing papers shows any awareness of the retraction. Yet most papers of the 2015 network have been published well after the retraction was published. There are two exceptions. The first [37], an original contribution, calls the Narayan result “controversial” while citing [29]. The second [38], a review article, notes both [29] and the retraction itself.

The rapid expansion of the citation network generated by the Narayan paper is remarkable. It must be ascribed to its subject and to the fact that it appeared in *Nature*. Moreover, its exposure was enhanced by [36] in the the *Reviews and Comments* section of the same *Nature* issue. This perhaps also explains why almost half of the primary citations are review articles. It is also evident that every review except one summarises the main finding of the Narayan paper as a matter of fact. This is significant because being cited in a review is considered the first step in canonisation of new knowledge, see also [14].

The Narayan paper is not only cited by reviews but also by original contributions. The citation is used as background knowledge in the Introduction section or as relevant evidence in the Discussion section of the paper. We have found that in this group of papers, diverse aspects of the work reported in the Narayan paper are cited. In one case [39], part of the experimental method of Narayan and co-authors is cited. This raises the interesting question whether retraction of a paper also means that its experimental methods have to be removed from the annals of science.

In both citation networks, we have looked for papers that directly cite the Narayan paper while one of its co-authors is also a co-author of the Narayan paper. We have identified two such papers in the 2015 network: [40] (published December 8, 2013, corrected December 16, 2013, and an erratum dated February 2014) and [41] (published online February 11, 2014). The dates of publication, respectively correction, are quite close to the date of retraction of the Narayan article, which is February 27, 2014. In neither paper, nor in the correction and erratum to the first, can one find any indication of the impending retraction.

The July 2015 search in Scopus of papers published after 2011 that match the search term sirt* AND necro* yielded 391 articles. (Precisely the same search on PubMed on the same day yielded 120 articles.) We checked for the presence of the Narayan paper, its retraction, and [29]: all three are present in the search result. For the overlap with the 2014 and 2015 citing collections, see Table 1. Obviously, the 2014 overlap is a subset of the 2015 overlap. The 2015 overlap has 10 papers that indirectly cite the Narayan paper: [42–45] (the only one also overlapping with the 2014 collection), [46] (in Chinese), [47–51]. We read all 10 papers. Of these, only one paper contains a passage that might refer to the Narayan paper without citing it ([43], p. 91):

- “Previous studies have also indicated that SIRT2 is a mediator of cell death. In particular, SIRT2 inhibition was shown to decrease the injury in cellular and animal models of PD and HD [2].”

The only citation that accompanies this statement (“[2]”, which corresponds to our reference [52]), occurs in neither of the two citing collections for the Narayan paper. The passage we quote cannot plausibly count as a reference to the Narayan paper. The result of the inspection of papers that indirectly cite the Narayan paper thus is zero.

### Discussion

Even when a paper has been retracted, it can be cited in good faith. Citing a paper before it is retracted is of course done in good faith. There is normally a time gap between publication of an article and its retraction. In one exceptional case, the gap was 24 years; the paper was still cited at that time [18].

**Fig. 3:**
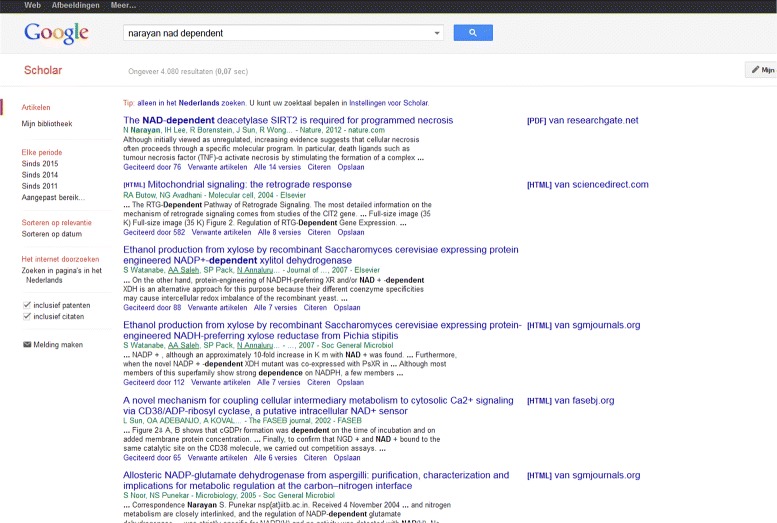
Screenshot of a Google Scholar search. The search was performed in March 2015 and used the keywords at the *top*. The Narayan paper is first on the list. Clearly, it is not marked as being retracted. Also the retraction, even though it has exactly the same title preceded by the word “Retraction”, is not among the results shown. It does turn up on the second page, however (not shown here)

The author who wants to avoid citing a paper that has been retracted will experience difficulties in finding out about the retraction [11]. Moreover, although *Nature* has put the word “RETRACTED” in capitals and red print on every digital page of the Narayan paper once the retraction was a fact, a researcher may have recourse to the hard-copy issue of *Nature* or may have added the digital paper to a private collection before it was retracted [53]. In our experience, none of the popular search engines Google Scholar, Scopus and Web of Science add a warning to a retracted paper in their list of search results. PubMed does, but in our experiments, PubMed was seen to retrieve far fewer documents than Scopus did. Chen c.s. provide screenshots to claim that Google Scholar explicitly marks retracted papers [25]. Our screenshot, Fig. 3, shows that this is not done consistently. That particular Google Scholar search was performed more than a year after publication of the retraction and the title of the retraction is identical to that of the original article except for the one word “Retraction”, the retraction itself does not occur among the first six hits. (In fact, it did not even occur on the first page of search results.) In search engines in general, searching on title or author may or may not turn up the retraction in the result list, and if it does, adding a year of publication to the search criteria is almost always sufficient to hide the retraction. Also, search engines have inevitable latency. In March, 2014, at least Scopus did not list the retraction in its search results when searching on “Narayan”, “NAD-dependent” and “deacetylase”. In September, 2014, the retraction was there. See also [54] for a discussion on search engines and retractions.

Finding out about a retraction becomes even more difficult when we do not look for entire articles but for passages instead. Modern information retrieval research investigates so-called *passage retrieval*, the retrieval of relevant passages rather than entire articles ([55], ch. 13). A paper is always retracted as a whole even though parts of it may be unaffected by the reasons for retraction. To be useful for practising scientists, a passage retrieval search engine will have to incorporate provisions for retrieving the retracted status of the paper from which the passage stems. To enable search engines to do this, publishers will have to make the status known in a structured way readable by a computer programme.

The very least that can be done is keeping track of retractions. The *Retraction Watch* blog does invaluable service here but it is not yet available for automated methods. Recently, the blog announced it had received a grant to set up a database of retractions [56]. PubMed explicitly marks retractions. If this is done in a machine-readable way, PubMed’s retraction list is a good starting point for a database of retractions. Also, the commercial service CrossMark by CrossRef promises to keep its users informed of retractions [57]. CrossMark relies on the voluntary participation of publishers, and although the current list of participating publishers is impressive, it is by no means complete. We feel that journal publishers should have done this long ago and for free because they publish both the original articles and their retractions.

With or without a database of retractions, it is feasible to automatically construct a citation network for a retracted paper. After all, any citation network is a subgraph of the graph defined by the citation relations identified by Scopus, Web of Science or Google Scholar. The programme can be written such that it continually monitors bibliographic descriptions added to the database to keep the network up-to-date. It would be interesting to find out the extent to which the published literature is citing retracted papers either directly or through a citation chain. The two citation networks for the Narayan paper suggest that the proportion of papers that occur in the citation network of a retracted paper may be a lot higher than we would think. Our research suggests that we can concentrate on directly citing articles to find propagation of a retracted result. With current, off-the-shelf passage retrieval techniques, it is possible to extract the citing passages in such articles automatically.

Authors of a paper published previously should be warned when one of their citations gets retracted. To be feasible, a publicly accessible database of retractions is a prerequisite. Authors should be given the opportunity to revise their paper if they think their conclusions are affected by the retraction. At the very least, they may want to flag the offending citation as being retracted.

Where automatic construction of citation trees is eminently feasible, assessing propagation beyond the primary citation in an automatic way is far more complicated, if possible at all. One possible route towards such a system exploits a proposal by Anicich to annotate every item in the list of references with markers indicating whether the citation supports the work, contradicts it, and so on [58]. Proponents of replacing normal text by hypertext documents have proposed similar markers for the relations connecting pieces of text, see for example [59].

A more thorough analysis would involve reasoning about the content of a paper. This presupposes that we have been able to translate what the paper says into a language that can be manipulated by a computer programme. Such a language is called a (knowledge) representation language ([60], ch. 12). Progress has been made in having a programme prepare such a translation (see, for example, [61]), but we are far from able to capture the relevant parts of what a text says. Complex sentences, anaphora and modalities (“we believe”, “we think”, “it is plausible”, and so on) all pose difficulties that have not yet been solved for routine use. It is not clear at which timescale these issues are solved to the extent that automatic assessment of damage done by a retracted paper is possible.

## Conclusions

To conclude, in line with what earlier authors have found [16–18, 26], propagation of retracted results through directly citing articles is a real scenario. On the other hand, in our case study, we have not seen propagation of a retracted result beyond those directly citing articles. Our result suggests that in this case authors display proper citing behaviour. More specifically, authors who publish about the relation between sirtuin-1 and necroptosis will cite the Narayan paper. In our study, such authors will therefore end up in the list of directly citing articles. This is aided by the fact that the Narayan paper is highly visible. It is published in *Nature* and moreover has an editorial comment that draws readers’ attention to it. Although a single case study can of course never rule out that retracted results propagate through articles with indirect citations (the scenario of [25]), we think that in environments with accessible literature and proper citing behaviour, spreading of retracted results through indirectly citing articles is not a probable event. In other words, the high visibility of a document published in a top-ranked journal makes it probable that results are spread but the results can be linked to their source. Documents published in a low-tier journal, on the other hand, will not be very visible. Therefore, one may speculate, such results do not spread out so quickly but if they do, the link to their source may be lost. In all this, the citing behaviour in a scientific community is a key factor.

In the search for automated support for handling retractions, there appear to be two extremes, neither of which is attractive. One extreme is handling fully by hand, which is impossible because the amount of labour involved is prohibitively large. The other extreme, handling retractions fully automatically, is currently infeasible and will remain so for some time to come. We therefore propose an approach that utilises the best of both worlds: a highly interactive computer programme operated by domain experts. The computer is good at following citation chains and highlighting passages in which a primary citation occurs, while the domain expert is good at judging the impact of retracted results. Modern computing environments involving highly interactive, very large displays enable the expert to view a large amount of information simultaneously. When a lot of material is collected this way, we may perhaps be able to answer questions such as the following: how many generations of citation must be followed before we can safely ignore citations even further away; is the influence of review articles indeed greater than that of original contributions; and, most importantly, are there original contributions of which conclusions have to be retracted because they crucially rely on assumptions that have been retracted? Finally, even for a single paper like the Narayan paper, following all citation chains is a lot of work. It seems only worthwhile if the results can be shared. A further question thus is how the results of such an exercise should be communicated.

## Additional files

Additional file 1 All texts with citing passages. PDF file. For every article that directly cites the Narayan article, this supplement lists all passages in which such a citation occurs. (PDF 115 kb)

Additional file 2 Specification of the 2014 citation network. dot file [33] that contains a complete specification of the 2014 citation network. A visual rendering of this network is provided as Additional files 4 and 5. The nodes are labelled with their BibTE X identifiers, which are expanded into full references in Additional file 6. (TXT 9.23 kb)

Additional file 3 Specification of the 2015 citation network. dot file [33] that contains a complete specification of the 2015 citation network. As above. (TXT 80.4 kb)

Additional file 4 The 2014 citation network with named nodes. PDF file showing the graph that constitutes the 2014 citation network with nodes labelled with their BibTE X identifiers (expanded in Additional file 6). In order to keep the figure readable, a large subgraph has been collapsed into the node “KaczmarekTree”. The latter node is expanded in Additional file 5. The figure is scalable and can be enlarged in any PDF viewer capable of zooming without becoming fuzzy. (PDF 62.1 kb)

Additional file 5 Subgraph of the 2014 citation network with named nodes. PDF file showing the subgraph with named nodes of the 2014 citation network that was collapsed into the node “KaczmarekTree” in Additional file 4. The properties of this figure are as for Additional file 4. (PDF 57.7 kb)

Additional file 6 Bibliographic references. BibTE X file with all bibliographic references to expand the labels that occur in the two dot files and in the graphs with named nodes. (BIB 695 kb)
