# Supplementary material for: Propagation of errors in citation networks: a study involving the entire citation network of a widely cited paper published in, and later retracted from, the journal Nature
Source: Res Integr Peer Rev. 2016 May 3;1:3. doi: 10.1186/s41073-016-0008-5 (PMC5793988; doi:10.1186/s41073-016-0008-5)
Supplement: Supplementary file 1 — All texts with citing passages. PDF file. For every article that directly cites the Narayan article, this supplement lists all passages in which such a citation occurs. (PDF 115 kb) [file 41073_2016_8_MOESM1_ESM.pdf]

Supplement to
Propagation of errors in citation networks: A study involving the entire citation network of a widely cited paper published in, and later retracted from, the journal Nature

Paul van der Vet* Harm Nijveen†

Contents

1 Introduction **1**

2 Results for the 2014 citing collection **1**
2.1 Reviews 2
2.2 Original contributions 3
2.3 Note 5

3 Results for the 2015 citing collection **5**
3.1 Reviews 6
3.2 Original contributions 7
3.3 Note 8

4 Bibliography **8**

1 Introduction

This supplement provides additional information about the citing collection formed by Narayan et al. [2012]. For all directly citing articles it contains a literal transcription of every text fragment in which Narayan et al. [2012] is cited in Sections 2 and 3. As explained in the main text, those articles are divided over the 2014 and 2015 collections with accompanying networks. The official retraction [Narayan et al., 2014] is absent from the 2014 set on the ground that, at the time, Scopus had not yet included this paper in their list of papers citing Narayan et al. [2012]. Also the paper that prompted the retraction, Newton et al. [2014], has been left out.

2 Results for the 2014 citing collection

We list the quotations, preceded by the number of the reference to Narayan et al. [2012] if the references are numbered. The quotation or quotations are enclosed in double quotes and are followed by a page number if page numbers are defined, or by "(e-pub)" of a digital-only publication of a journal that numbers articles rather than pages.

*Human Media Interaction Group, University of Twente, Enschede, the Netherlands and ZGT Academy, Ziekenhuisgroep Twente, Almelo and Hengelo, the Netherlands

†Bioinformatics Laboratory and Seed Lab, Laboratory of Plant Physiology, both at Wageningen University, Wageningen, the Netherlands

2.1 Reviews

Cha and Kim [2013] (ref. 39) "In the most recent study, Sirt2 was shown to play a pivotal role in programmed necrosis through the deacetylation of Receptor-Interacting Protein 1 (RIP1), and that Sirt2 inhibitor may be useful as a novel therapy in ischemic stroke and myocardial infarction (39)." (p. 430)

Chakraborty and George Priya Doss [2013] (ref. 133) "Another study with Sirt2 -/- mice or wild-type mice, has shown that management with particular inhibitor of SIRT2 of the hearts of Sirt2 -/- mice or wild-type mice, can protect against ischaemic injury. Taken together, these results suggest that SIRT2 act as an important regulator of programmed necrosis. It is also pointed out that these deacetylase inhibitors might represent an original approach to defend against the diseases such as necrotic injuries, including ischaemic stroke and myocardial infarction [133]." (p. 670)

Christofferson et al. [2014] (ref. 103) "SIRT2, a NAD-dependent cytosolic deacetylase that removes acetyl groups from their substrates, is required for necroptosis (103). Treatment with a SIRT2 deacetylase inhibitor, AGK2, can block RIP1-RIP3 complex formation and cell death after necroptotic stimulation, and *Sirt2*$^{-/-}$ cells are resistant to necroptosis. SIRT2 binds to the RHIM domain of RIP3, but RIP1 is the substrate of SIRT2. SIRT2 promotes the deacetylation of K530 of RIP1, which is directly involved in interacting with RIP3 and is adjacent to the RIP1 RHIM domain. RIP1 is basally acetylated, but the levels of acetylation are reduced in necroptotic cells. Although RIP1 κ530A, a constitutively deacetylated mutant, forms a complex with RIP3, such formation is not sufficient for the cells to undergo necroptosis (103). Thus, the functional significance of complex IIb formation may be for RIP3 to deliver SIRT2 to RIP1 to mediate the deacetylation of RIP1." (p. 137)

Giampietri et al. [2014] (ref. 20) "Recently the activity of the NAD-dependent deacetylating enzyme SIRT2 has been found to be implicated in the RIP1-mediated recruitment of RIP3 and the necrosome formation [20]." (e-pub)

Kaczmarek et al. [2013] "The activity of the NAD-dependent deacetylating enzyme SIRT2 (Narayan et al., 2012) has been implicated in the RIPK1- mediated recruitment of RIPK3 and the formation of complex IIb (Figure 1)." (p. 210; in the figure, Narayan et al. [2012] does not appear)

Li et al. [2013a] "Interestingly, recent studies found that programmed necrosis has relationships with the NAD-dependent deacetylase SIRT2 (Narayan et al., 2012). SIRT2 binds constitutively to the C-terminal RHIM domain of RIP3. However, it is not clear whether the RHIM domain is sufficient for the interaction between the two proteins. Deletion or siRNA knockdown of SIRT2 can block the formation of the RIP1/RIP3 complex in mice. SIRT2 regulates RIP1 acetylation via deacetylation at Lys530 of RIP1, which promotes RIP1/RIP3 complex formation in TNF-induced necrosis. When SIRT2 is inhibited by a specific pharmacological inhibitor AGK2, ischemic injury in the heart and the brain is reduced in mice (Narayan et al., 2012)." (p. 146–147)

Liddy et al. [2013] (ref. 70) "SIRT2 deacetylase inhibition is protective against I/R injury, because SIRT2 binds receptor interacting protein-3 (RIP3), which is part of a necrosis-promoting complex stimulated by tumor necrosis factor α [70]." (e-pub)

Martínez-Redondo and Vaquero [2013] (ref. 175) "Other remarkable sirtuin substrates are cyclophilin D and RIP1. Both are involved in necrotic cell death but are activated by the deacetylation activity of SIRT3 and SIRT2, respectively.[175,176]" (p. 157)

Menzies and Auwerx [2013] "Recent evidence has also linked SIRT2 with a potential role in cardiac protection from ischemic injury (Narayan *et al.* 2012)." (p. 102)

Morgan and Liu [2013] (ref. 83) "The RIP1-RIP3 complex formation is also apparently negatively regulated by the acetylation of RIP1 on lysine 530, which is near its RHIM domain, and thus, the deacetylation of RIP1 must be carried out by the RIP3-binding deacetylase SIRT2 for necrosis to proceed (83)." (p. 264)

Moriwaki and Chan [2013] "A recent study found that RIP1 was acetylated at Lys530 (Narayan et al. 2012). Deacetylation by sirtuin2 (SIRT2), which constitutively binds to RIP3, facilitated TNF-induced necrosis. These results suggest that one of the early events that stabilize the RIP1–RIP3 necrosome could be SIRT2-mediated deacetylation of RIP1. More work is required to confirm the role of SIRT2 and RIP1 acetylation in physiological necrosis." (p. 1643)

Mouchiroud et al. [2013] "Furthermore, SIRT2 deacetylates phosphoenolpyruvate carboxykinase to control gluconeogenesis (Jiang *et al.*, 2011) and was recently shown to deacetylate the receptor-interacting protein-1, and thereby serve as a critical component of the tumor necrosis factor α-mediated programmed necrosis pathway (Narayan *et al.*, 2012)." (p. 399)

Nikoletopoulou et al. [2013] (ref. 130) "Recent findings indicate that SIRT2 is also involved in the regulation of necroptosis [130]. SIRT2 associates with RIP3 and mediates RIP1 deacetylation in response to TNFα stimulation. As a consequence, RIP1 and RIP3 directly interact and form complex II, triggering necroptosis [130]. These early observations suggest a functional link between autophagy and necroptosis. However, much still remains to be uncovered about the molecular mechanisms underlying the complex interplay between these two processes." (p. 3456)

Starke et al. [2014] (ref. 18) "TNF-α binding to the more physiologically active of its receptors, TNF receptor 2, also results in inflammation and apoptosis via activation of NFκB and cFos/Jun-induced factors (Fig. 1) [15–18]." (p. 270)

"TNF-α also plays an important role in inflammatory states through its role as a facilitator of apoptosis, via upregulation of the Fas-associated death domain (FADD) pathway [15], and necrosis and necroptosis through the receptor-interacting protein pathway and production of cathepsin and reactive oxygen species [15, 18, 22]." (p. 270)

Webster et al. [2014] (ref. 52) "Sirt2 also has nuclear and cytosolic targets, and in contrast to Sirt1, the depletion of Sirt2 has ameliorative effects against multiple stressors [50-52]." (p. 526)

Wu et al. [2014] "Mechanisms of tumor suppression by sirtuins initially focused on their ability to halt the cell cycle, inactivate oncogenic transcription factors, and promote DNA repair, but more recent studies have shown that their effects on energy metabolism may be equal, if not more important, for tumor suppression (Figure 2; Csibi et al., 2013; Finley et al., 2011; Firestein et al., 2008; Herranz et al., 2010; Jeong et al., 2013; Kim et al., 2010; Narayan et al., 2012; Oberdoerffer et al., 2008; Sebastián et al., 2012; Serrano et al., 2013)." (p. 14) (In Figure 2, SIRT2 does not appear and, hence, the Narayan paper is not relevant for the Figure.)

"There is evidence that SIRT2, the cytosolic sirtuin, is also a tumor suppressor. Deletion of *Sirt2* results in spontaneous tumorigenesis in the liver and accelerates the 7,12-dimethylbenz(a)anthracene (DMBA)/12-O-tetradecanoylphorbol-13-acetate model of skin cancer (Narayan et al., 2012; Serrano et al., 2013). One mechanism is likely to be cell cycle control, as SIRT2 deacetylates and regulates CDH1 and CDC20, members of the anaphase-promoting complex (Narayan et al., 2012)." (p. 15)

"*Sirt2* deletion causes spontaneous tumorigenesis in mice (Narayan et al., 2012)" (p. 15, in the table)

Yu et al. [2013] (ref. 54) "Acetylation of RIPK1 lysine 530 by NAD-dependent deacetylase sirtuin (SIRT2) modulated RIPK1–RIPK2 complex formation and TNF-α-stimulated necroptosis [54]." (p. 885)

Yuan et al. [2013] (ref. 242) "RIP1 is required for programmed necrosis[242]" (in table 2 on p. 1402)

2.2 Original contributions

Bauer et al. [2013] (ref. 62) "Another example where none of the applied VS tools could obtain good benchmark results was the NAD-dependent deacetylase sirtuin-2 (SIRT2). The inhibition of SIRT2 was recently reported to have neuroprotective function.[61] Narayan et al. also showed that SIRT2

was involved in the regulation of necrosis.[62] This highlights the importance of SIRT2 as a promising target." (p. 1456) (This paper is about a software tool and sirtuins are just an example.)

Cai et al. [2014] (ref. 13) "Other proteins including CYLD and SIRT2 are also suggested to play a role in the formation of the necrosome[12,13]." (p. 55, Introduction)

Disch et al. [2013] (ref. 12) "Modulating sirtuin activity,[3] either through overexpression (activation) or knockdown (inhibition), has been proposed to be beneficial in numerous disease states including those related to metabolism,[4] cancer,[5,6] neurodegeneration,[7−−9] inflammation,[10,11] and ischemic injury.[12]" (p. 3666, Introduction)

Kragh et al. [2014] "The increased IκBα protein in our MSA mouse model could also represent a response to oxidative stress that has been demonstrated to inhibit the ubiquitination of phosphorylated IκBα otherwise destined for degradation (Kalita et al., 2011; Narayan et al., 2012). Irrespective of mechanism, our results suggest the strong reduction of NF-κB activation in α-syn expressing SH-SY5Y cells could be the result of an increased IκBα expression (Yuan et al., 2008)." (p. 180, Discussion)

"SIRT2 dependent deacetylation of RIP-1 kinase changes TNF-R signaling from an activator of protective NF-κB signaling to an inducer of necrosis (Narayan et al., 2012). SIRT2 inhibition is neuroprotective in some PD models (Outeiro et al., 2007) and α-syn aggregate-dependent sensitization to SIRT2 activity could play a functional role in oligodendroglial MSA models where FAS dependent signaling is turned into a cytotoxic path (Kragh et al., 2013)." (p. 182, Discussion)

Li et al. [2013b] (ref. 23) "However, opposing evidence show that Sirt2 absence sensitizes cells to apoptotic stimuli via regulating p53 [21,22]. Recently, Sirt2 was evidenced in the process of necrosis via binding to RIP3 and deacetylating RIP1 [23]. Those works also strongly challenge the work of He et al. [13]." (p. 665, Discussion])

Li et al. [2013c] (ref. 10) "For examples, a latest study has indicated that SIRT2 mediates TNF-α-induced programmed necrosis and myocardial ischemia-reperfusion injury [10]" (p. 36, Introduction)

"Some studies have also indicated that SIRT2 is amediator of cell death, whichmaybecomea therapeutic target for such diseases as PD and myocardial ischemia: A latest study has indicated that SIRT2 plays a key role in TNF- α-induced programmed necrosis and that AGK2 was shown to attenuate myocardial ischemia-reperfusion injury [10]" (p. 39, Discussion)

Li et al. [2013d] (ref. 6) "The function of Sirtuin family proteins is complex, which includes regulation of the oxidative stress, cell survival, metabolism, aging and longevity [4-6]." (p. 9, Introduction)

"Recent data have revealed another important biological function of SIRT2, namely a regulator of necroptosis[6]." (p. 12, Discussion)

Nomura et al. [2014] (ref. 38) "Although little is known aboput how this complex formation is regulated, it was recently reported that RIP1–RIP3 complex formation requires RIP1 deacetylation by NAD-dependent deacetylase SIRT2 (38)." (p. 1064, Discussion)

Pais et al. [2013] "The receptor-interacting protein 1 (RIP-1), which is also involved in inflammatory signalling pathways, was also found to be a SIRT2 substrate. The deacetylation of RIP-1 by SIRT2 stabilizes the RIP-1–RIP-3 protein complex required for TNF-induced cell death in fibroblasts (Narayan *et al*, 2012). Altogether, these results provide evidence that SIRT2 deacetylates important regulators of inflammation such as NF-κB and RIP-1." (p. 2604, Introduction)

"The increased caspase-3 activity and apoptosis in SIRT2-deficient cells contrast other findings obtained in fibroblasts where reduction in SIRT2 levels or activity was protective towards TNF-induced cell death (Rothgiesser *et al*, 2010a; Narayan *et al*, 2012). Curiously, the SIRT2 inhibitor AGK2 was recently shown to induce apoptosis in another microglial cell line, BV2 (Li *et al*, 2013)." (p. 2612, Discussion)

"This could be because NF-κB activation through TLR3, contrary to the other TLRs, is dependent on RIP-1 (Meylan *et al*, 2004), another recently identified target of SIRT2 (Narayan *et al*, 2012)." (p. 2612, Discussion)

Petrilli et al. [2013] (ref. 14) "SIRT2 is mainly cytoplasmic and its known substrates include: α-tubulin, partitioning defective 3 homolog (PAR3), p53, K-RAS, histone H4K16, forkhead Box O1 and 3a (FOXO1 and 3a) and RIP1 [9-14]." (p. 2354–2355, Introduction)

"Additionally, tumor necrosis factor alpha (TNF-α) was shown to activate necroptosis via deacetylation of receptor-interacting protein 1 (RIP1) by receptor-interacting protein 3 (RIP3) bound SIRT2 allowing the formation of a stable complex in L929 and Jurkat T cells [14]." (p. 2359, Discussion)

Ramakrishnan et al. [2014] (ref. 21) "Sirt2, the primary cytoplasmic sirtuin, has been attributed tumor suppressor functions and a role in maintaining genome integrity as well as a role in programmed necrosis (20-22)." (p. 6055, Introduction)

Sayd et al. [2014b] (ref. 14) "More recently another member of the sirtuin family, SIRT2, has got much attention due to its involvement in programmed necrosis [14]." (p. 104, Introduction)

"A very recent report has noteworthy placed SIRT2 at the core of programmed necrosis [14]. In GSCs, blockade of SIRT2 expression or enzymatic activity did not counteract resveratrol-induced necrosis, suggesting that in that case necrosis is likely the result of a disordered catastrophic response of the cells to severe energy depletion." (p. 111, Discussion)

Taes et al. [2013] (ref. 7) "However, these enzymes have several other functions in the cell. For instance, Sirt2 has recently been suggested to play a role in programmed necrosis (7)." (p. 1783, Introduction)

"Furthermore, as Sirt2 has been recently shown to play a pivotal role in programmed necrosis (7), our data indicate that this type of cell death does not contribute to the process of neurodegeneration such as seen in the $SOD1^{G93A}$ model of ALS." (p. 1788, Discussion)

Vitner et al. [2014] (ref. 45) "Paraffin sections were incubated with an antibody to Ripk3 (ref. 45) [...] rabbit antibody to RIPK3 (ref. 45) [...]" (online methods, no page number)

Wu et al. [2013] (ref. 34) "In the effort to seek novel molecules involved in necroptosis, NAD-dependent deacetylase sirtuin-2 (SIRT2), phosphoglycerate mutase family member 5 (PGAM5) and mixed lineage kinase domain-like protein (Mlkl) have been recently revealed as key signaling factors in necroptosis [18, 32, 34-36]." (p. 995, Introduction)

Yamagata et al. [2014] "For instance, the SIRT2-catalyzed deacetylation of a-tubulin (Inoue et al., 2007; North et al., 2003) and histone H4 (North et al., 2003; Vaquero et al., 2006) functions in the regulation of cell-cycle progression, and its deacetylation of receptor-interacting protein 1 (RIP1) is responsible for the modulation of RIP1-RIP3 complex formation and thus regulates programmed necrosis (Narayan et al., 2012)." (p. 345, Introduction)

Yan et al. [2013] (ref. 17) "By contrast, the reperfused MI affected primarily the epicardial layer while the endocardial layers were spared [14-18]." (p. 29, Results)

2.3 Note

Zhou and Yuan [2012] is mentioned here for completeness only. The note is in fact a summary of the Narayan paper and there is no sense in picking out passages that specifically address the Narayan paper.

3 Results for the 2015 citing collection

We list here those papers that are not already member of the 2014 citing collection.

3.1 Reviews

Bae et al. [2014] "Moreover, it has been suggested that cylindromatosis (CYLD) and sirtuin-2 (SIRT2) are responsible for necroptosis (Moquin et al. 2013; Narayan et al. 2012)." (p. 692)

"Amid efforts to understand the role of sirtuin-2 (SIRT2), RIP3 was serendipitously revealed as a candidate protein of interacting with it through immunoprecipitation and mass spectroscopic analyses (Narayan et al. 2012)." (p. 692)

"Also, a well-known SIRT2 inhibitor AGK2 was employed as a pharmacological inhibitor to confirm the engagement of SIRT2 in TNFα-mediated programmed necrosis (Narayan et al. 2012)." (p. 694)

Cho [2014] (ref. 21) "It has also been published that the NAD-dependent deacetylase, SIR2, is involved in the regulation of TNF-mediated programmed necrosis (21). It not only recruits RIP3, but also catalyzes the deacetylation of RIP1 to allow it to be in a stable conformation, forming a necrotic complex." (p. 1402)

Jouan-Lanhouet et al. [2014] (ref. 62) "On the other hand, necrosome formation and necroptosis induction appear to be positively regulated by CYLD, SIRT2, RIPK3 kinaseactivity and MLKL [61–63], but the role of SIRT2 was recently questioned [64]." (p. 4) Reference 64 is Newton et al. [2014].

Lee et al. [2014] "SIRT2 is located primarily in the cytoplasm and has functions in cell cycle regulation, oligodendrocyte differentiation, and programmed cell death (Dryden et al., 2003; Li et al., 2007b; Narayan et al., 2012)." (p. 26)

Nührenberg et al. [2014] "Although Sirt4-*null* and Sirt5-*null* mice lack cardiac alterations (Haigis et al. 2006; Lombard et al. 2007), Sirt1, Sirt2, Sirt3, and Sirt7 are indispensable for cardiac development and function (Cheng et al. 2003; Narayan et al. 2012; Sundaresan et al. 2009; Vakhrusheva et al. 2008)." (p. 591–592)

"[Sirt2] Protects heart from ischemic injury (Narayan et al. 2012)" (p. 594, Table 3)

Roth and Chen [2014] (ref. 140) "Most recently, SIRT2 is shown to regulate programmed necrosis by targeting receptor-interacting proteins.[140]" (p. 1614)

Sayd et al. [2014a] (ref. 25) "De plus, SIRT2 déacétyle la sérine/thréonine kinase RIP1 (receptor-interacting protein 1) au niveau de la lysine 530 [25]. Il en résulte la formation du complexe RIP1-RIP3 qui active la mort cellulaire par nécrose en réponse au facteur TNF-α (tumor necrosis factor-α). Sur la base de ces différentes observations, la stimulation de l'activité de SIRT2 pourrait constituer la base de nouvelles approches thérapeutiques pour induire la nécrose de cellules tumorales." (p. 534)

Translation (by the current authors): "Moreover, SIRT2 deacetylates the serine/threonine kinase RIP1 [...] at Lys530 [25]. This results in the formation of the complex RIP1-RIP3 which activates cell death through necrosis in response to TNF-α. On the basis of these different observations, the stimulation of the activity of SIRT2 may constitute the basis of new therapeutic approaches for inducing necrosis of tumor cells."

Shao et al. [2014] (ref. 74) "It is reported that Sirt2 inhibition prevents formation of receptor-interacting protein 1 (RIP1) to receptor-interacting protein 3 (RIP3) complex required for programmed necrosis and reduces myocardial ischemia/reperfusion injury[74], but this result has been questioned recently[75]. The same group retracted this study owing to failure of reproducing an in vitro requirement for Sirt2 in TNF-α-mediated necroptosis, but Sirt2 and RIP3 were again confirmed to interact and it was believed that the absence of Sirt2 protects against ischemic myocardial injury[76]." (p. 655) These authors are aware of both Newton et al. [2014] (their ref. 75) and the retraction itself (their ref. 76).

Van Meter et al. [2014] (ref. 44) "Less is currently known about the remaining mammalian sirtuin genes. SIRT2 is predominantly localized in the cytoplasm, but can shuttle to the nucleus under stress. In the cytoplasm SIRT2 deacetylates RIP1 to promote programmed necrosis among other known functions [44]." (p. 183)

Wagner and Hirschey [2014] "However, sirtuin ablation is not always pathogenic. For example, mice lacking SIRT2 are markedly protected from ischemic injury (Narayan et al., 2012)." (p. 5)

3.2 Original contributions

Buler et al. [2014] (ref. 56) "Because of these apparently conflicting findings reported in the literature, the significance of SIRT2 induction for metformin action on glucose tolerance is currently difficult to assess. Furthermore, SIRT2 maintains genome integrity, represses tumor formation, and is necessary for programmed necrosis (56, 57). Consequently, SIRT2 induction corresponds well with the antitumor properties of metformin (58, 59)." (p. 3234, Discussion)

Chen et al. [2015] (ref. 18) "A role for SIRT2 in mediating programmed necrosis, and a possible amelioration of necrotic injuries, including those that result from ischemic stroke and myocardial infarction, by inhibition of SIRT2 enzyme activity has been proposed but remains controversial [18, 19]." (Results, e-pub)

"Although our preclinical efficacy test of AK7 in a mouse model of ALS yielded negative results, this finding is consistent with the less defined and potentially conflicting roles of SIRT2 in ALS [17]. Moreover, treatment with AK7 was not beneficial in mouse model of stroke, where a therapeutic role of SIRT2 inhibition has been proposed but remains controversial [18, 19]." (Discussion, e-pub)

These authors were aware of the paper by Newton et al. [2014] but not of the actual retraction.

Nie et al. [2014] (ref. 6) "SIRT2 has been shown to play seemingly paradoxical roles in cell survival: SIRT2 has been indicated as a key mediator of programmed necrosis [6]; and SIRT2 inhibition has been shown to produce beneficial effects in models of Parkinson's disease (PD) and Huntington's disease (HD) [7, 8]." (p. 166, Introduction)

"Multiple studies have also suggested contrasting roles of SIRT2 inhibition in cell death under various conditions: SIRT2 inhibition has been shown to produce beneficial effects in models of PD, HD [7, 8] and ischemic myocardial damage [6]." (p. 169, Discussion)

(The references 7 and 8 are not relevant for the present research.)

Pan et al. [2014] (ref. 38) "Alternatively, to confirm the necroptosis during HIV-1 infection, cell viability assay, which has been used by other groups, was used and also confirmed the HIV-1-induced necroptosis [38,39] [...]." (e-pub, Results)

Pantazi et al. [2014] (ref. 54) "[...] sirtinol is less potent to prevent the protection provided by PC [ischemic preconditioning]. This fact may be attributed to its additional inhibitory effect on SIRT2; given that in recent studies, inhibition of SIRT2 has been found to be protective [54,55], the results obtained after sirtinol treatment might be the consequence of the inhibition of both SIRT2 (possible protective effect) and SIRT1 (detrimental effect)." (p. 500, Discussion)

Qiao et al. [2014] (ref. 27) "The hearts of SIRT2 knockout mice, and wild-type mice treated with a specific pharmacological inhibitor of SIRT2, show marked protection from ischaemic injury [27]." (p. 383, Discussion)

Vieira et al. [2014] "While the precise mechanism by which RIP1 and RIP3 induce necroptosis is not fully understood it is known that their kinase activity is important for this process (Cho et al., 2009; He et al., 2009; Zhang et al., 2009) and recently it was shown that their interaction is regulated by sirtuin2 (SIRT2)-dependent RIP1 deacetylation (Narayan et al., 2012). Nevertheless, the events

that trigger the assembly of the necrosome in the context of cerebral ischemia are not yet known." (p. 27, Introduction)

"Necroptosis has been demonstrated to be a relevant mechanism of cell death both in neurons (Li et al., 2008; Wang et al., 2012) as well as non-neuronal systems (Smith et al., 2007; Upton et al., 2010; Linkermann et al., 2012; Narayan et al., 2012; Simenc and Lipnik-Stangelj, 2012)." (p. 31, Discussion)

Viringipurampeer et al. [2014] (ref. 44) "Most recently, SIRT2 has been shown to regulate the deacetylation of RIP1, which is required for stable RIP1-RIP3 complex formation through RIP homotypic interaction motifs, and this deacetylation is RIP3-dependent process.[44]" (p. 673, Discussion)

Yoo et al. [2015] (ref. 35) "In myocardial ischemia, the genetic and pharmacological inhibition of SIRT2 was shown to protect the heart from ischemic damage.[35]" (p. 75, Discussion)

3.3 Note

Parthasarathy et al. [2014] (ref. 11) "Narayan et al., in their previous reports, presented the role of SIRT2 which is an enzyme involved in mediating the complex formation. The activity of SIRT2 is dependent on its cofactor NAD^+ [11]. The lower levels of NAD^+ indicated higher acetylation of α-tubulin which further leads to increased perinuclear localization of mitochondria in cells. Yet, little is understood about the role of microtubules in mitochondria-mediated inflammasome activation." (p. 697–698)

4 Bibliography

J.H. Bae, J.-H. Shim, and Y.S. Cho. Chemical regulation of signaling pathways to programmed necrosis. *Archives of Pharmacal Research*, 37(6):689–697, 2014.

M.R. Bauer, T.M. Ibrahim, S.M. Vogel, and F.M. Boeckler. Evaluation and optimization of virtual screening workflows with DEKOIS 2.0 - a public library of challenging docking benchmark sets. *Journal of Chemical Information and Modeling*, 53(6):1447–1462, 2013.

M. Buler, S.-M. Aatsinki, V. Izzi, J. Uusimaa, and J. Hakkola. SIRT5 is under the control of PGC-1α and AMPK and is involved in regulation of mitochondrial energy metabolism. *FASEB Journal*, 28(7):3225–3237, 2014.

Z. Cai, S. Jitkaew, J. Zhao, H.-C. Chiang, S. Choksi, J. Liu, Y. Ward, L.-G. Wu, and Z.-G. Liu. Plasma membrane translocation of trimerized MLKL protein is required for TNF-induced necroptosis. *Nature Cell Biology*, 16(1):55–65, 2014.

Y.I. Cha and H.-S. Kim. Emerging role of sirtuins on tumorigenesis: Possible link between aging and cancer. *BMB Reports*, 46(9):429–438, 2013.

C. Chakraborty and C. George Priya Doss. Sirtuins family — Recent development as a drug target for aging, metabolism, and age related diseases. *Current Drug Targets*, 14(6):666–675, 2013.

X. Chen, P. Wales, L. Quinti, F. Zuo, S. Moniot, F. Herisson, N.A. Rauf, H. Wang, R.B. Silverman, C. Ayata, M.M. Maxwell, C. Steegborn, M.A. Schwarzschild, T.F. Outeiro, and A.G. Kazantsev. The sirtuin-2 inhibitor AK7 is neuroprotective in models of Parkinson's disease but not amyotrophic lateral sclerosis and cerebral ischemia. *PLoS ONE*, 10(1):e0116919, 2015.

Y.S. Cho. Perspectives on the therapeutic modulation of an alternative cell death, programmed necrosis (review). *International Journal of Molecular Medicine*, 33(6):1401–1406, 2014.

D.E. Christofferson, Y. Li, and J. Yuan. Control of life-or-death decisions by RIP1 kinase. *Annual Review of Physiology*, 76:129–150, 2014.

J.S. Disch, G. Evindar, C.H. Chiu, C.A. Blum, H. Dai, L. Jin, E. Schuman, K.E. Lind, S.L. Belyanskaya, J. Deng, F. Coppo, L. Aquilani, T.L. Graybill, J.W. Cuozzo, S. Lavu, C. Mao, G.P. Vlasuk, and R.B. Perni. Discovery of thieno[3,2-*d*]pyrimidine-6-carboxamides as potent inhibitors of SIRT1, SIRT2, and SIRT3. *Journal of Medicinal Chemistry*, 56(9):3666–3679, 2013.

C. Giampietri, D. Starace, S. Petrungaro, A. Filippini, and E. Ziparo. Necroptosis: Molecular signalling and translational implications. *International Journal of Cell Biology*, page 490275, 2014.

S. Jouan-Lanhouet, F. Riquet, L. Duprez, T. Vanden Berghe, N. Takahashi, and P. Vandenabeele. Necroptosis, in vivo detection in experimental disease models. *Seminars in Cell and Developmental Biology*, 35:2–13, 2014.

A. Kaczmarek, P. Vandenabeele, and D. Krysko. Necroptosis: The release of damage-associated molecular patterns and its physiological relevance. *Immunity*, 38(2):209–223, 2013.

C.L. Kragh, A.M. Gysbers, E. Rockenstein, K. Murphy, G.M. Halliday, E. Masliah, and P.H. Jensen. Prodegenerative IκBα expression in oligodendroglial α-synuclein models of multiple system atrophy. *Neurobiology of Disease*, 63:171–183, 2014.

J. Lee, D.-G. Jo, D. Park, H.Y. Chung, and M.P. Mattson. Adaptive cellular stress pathways as therapeutic targets of dietary phytochemicals: Focus on the nervous system. *Pharmacological Reviews*, 66(3):815–868, 2014.

J. Li, Q. Yin, and H. Wu. Structural basis of signal transduction in the TNF receptor superfamily. *Advances in Immunology*, 119:135–153, 2013a.

Y. Li, D. Dai, Q. Lu, M. Fei, M. Li, and X. Wu. Sirt2 suppresses glioma cell growth through targeting NF-κB-miR-21 axis. *Biochemical and Biophysical Research Communications*, 441(3):661–667, 2013b.

Y. Li, H. Nie, D. Wu, J. Zhang, X. Wei, and W. Ying. Poly(ADP-ribose) polymerase mediates both cell death and ATP decreases in SIRT2 inhibitor AGK2-treated microglial BV2 cells. *Neuroscience Letters*, 544:36–40, 2013c.

Z. Li, Q.R. Xie, Z. Chen, S. Lu, and W. Xia. Regulation of SIRT2 levels for human non-small cell lung cancer therapy. *Lung Cancer*, 82(1):9–15, 2013d.

K.A. Liddy, M.Y. White, and S.J. Cordwell. Functional decorations: Post-translational modifications and heart disease delineated by targeted proteomics. *Genome Medicine*, 5(2):20, 2013.

P. Martínez-Redondo and A. Vaquero. The diversity of histone versus nonhistone sirtuin substrates. *Genes and Cancer*, 4(3-4):148–163, 2013.

K. Menzies and J. Auwerx. An acetylation rheostat for the control of muscle energy homeostasis. *Journal of Molecular Endocrinology*, 51(3):T101–T113, 2013.

M.J. Morgan and Z.-G. Liu. Programmed cell death with a necrotic-like phenotype. *Biomolecular Concepts*, 4(3):259–275, 2013.

K. Moriwaki and F.K.-M. Chan. RIP3: A molecular switch for necrosis and inflammation. *Genes and Development*, 27(15):1640–1649, 2013.

L. Mouchiroud, R.H. Houtkooper, and J. Auwerx. NAD+ metabolism: A therapeutic target for age-related metabolic disease. *Critical Reviews in Biochemistry and Molecular Biology*, 48(4):397–408, 2013.

N. Narayan, I.H. Lee, R. Borenstein, J. Sun, R. Wong, G. Tong, M.M. Fergusson, J. Liu, I.I. Rovira, H.-L. Cheng, Wang G., M. Gucek, D. Lombard, F.W. Alt, M.N. Sack, E. Murphy, L. Cao, and T. Finkel. The NAD-dependent deacetylase SIRT2 is required for programmed necrosis. *Nature*, 492:199–204, 2012.

N. Narayan, I.H. Lee, R. Borenstein, J. Sun, R. Wong, G. Tong, M.M. Fergusson, J. Liu, I.I. Rovira, H.-L. Cheng, Wang G., M. Gucek, D. Lombard, F.W. Alt, M.N. Sack, E. Murphy, L. Cao, and T. Finkel. Retraction: The NAD-dependent deacetylase SIRT2 is required for programmed necrosis. *Nature*, 506:516, 2014.

K. Newton, J.M. Hildebrand, Z. Shen, D. Rodriguez, S. Alvarez-Diaz, S. Petersen, S. Shah, D.L. Dugger, C. Huang, J. Auwerx, P. Vandenabeele, D.R. Green, A. Ashkenazi, V.M. Dixit, W.J. Kaiser, A. Strasser, A. Degterev, and J. Silke. Is SIRT2 required for necroptosis? *Nature*, 506(7489):E4–E6, 2014.

H. Nie, Y. Li, C. Wang, X. Chen, B. Liu, D. Wu, and W. Ying. SIRT2 plays a key role in both cell cycle regulation and cell survival of BV2 microglia. *International Journal of Physiology, Pathophysiology and Pharmacology*, 6(3):166–171, 2014.

V. Nikoletopoulou, M. Markaki, K. Palikaras, and N. Tavernarakis. Crosstalk between apoptosis, necrosis and autophagy. *Biochimica et Biophysica Acta - Molecular Cell Research*, 1833(12):3448–3459, 2013.

M. Nomura, A. Ueno, K. Saga, M. Fukuzawa, and Y. Kaneda. Accumulation of cytosolic calcium induces necroptotic cell death in human neuroblastoma. *Cancer Research*, 74(4):1056–1066, 2014.

T. Nührenberg, R. Gilsbach, S. Preissl, T. Schnick, and L. Hein. Epigenetics in cardiac development, function, and disease. *Cell and Tissue Research*, 356(3):585–600, 2014.

T.F. Pais, É.M. Szego, O. Marques, L. Miller-Fleming, P. Antas, P. Guerreiro, R.M. De Oliveira, B. Kasapoglu, and T.F. Outeiro. The NAD-dependent deacetylase sirtuin 2 is a suppressor of microglial activation and brain inflammation. *EMBO Journal*, 32(19):2603–2616, 2013.

T. Pan, S. Wu, X. He, H. Luo, Y. Zhang, M. Fan, G. Geng, V.C. Ruiz, J. Zhang, L. Mills, C. Bai, and H. Zhang. Necroptosis takes place in human immunodeficiency virus type-1 (HIV-1)-infected $CD4^+$ T lymphocytes. *PLoS ONE*, 9(4):e93944, 2014.

E. Pantazi, M.A. Zaouali, M. Bejaoui, A. Serafin, E. Folch-Puy, V. Petegnief, N. De Vera, H.B. Abdennebi, A. Rimola, and J. Roselló-Catafau. Silent information regulator 1 protects the liver against ischemia-reperfusion injury: Implications in steatotic liver ischemic preconditioning. *Transplant International*, 27(5):493–503, 2014.

P.T. Parthasarathy, Y. Cho, R. Lockey, and N. Kolliputi. An old molecule with a new role: microtubules in inflammasome regulation. *Cell Biochemistry and Biophysics*, 70(1):697–698, 2014.

A.M. Petrilli, M. Bott, and C. Fernández-Valle. Inhibition of SIRT2 in merlin/NF2-mutant Schwann cells triggers necrosis. *Oncotarget*, 4(12):2354–2365, 2013.

W. Qiao, W. Zhang, Y. Gai, L. Zhao, and J. Fan. The histone acetyltransferase MOF overexpression blunts cardiac hypertrophy by targeting ROS in mice. *Biochemical and Biophysical Research Communications*, 448(4):379–384, 2014.

G. Ramakrishnan, G. Davaakhuu, L. Kaplun, W.-C. Chung, A. Rana, A. Atfi, L. Miele, and G. Tzivion. Sirt2 deacetylase is a novel AKT binding partner critical for AKT activation by insulin. *Journal of Biological Chemistry*, 289(9):6054–6066, 2014.

M. Roth and W.Y. Chen. Sorting out functions of sirtuins in cancer. *Oncogene*, 33(13):1609–1620, 2014.

S. Sayd, M.-P. Junier, and H. Chneiweiss. SIRT2, une déacétylase aux multiples talents. *Médecine/Sciences*, 30(5):532–536, 2014a.

S. Sayd, C. Thirant, E.A. El-Habr, J. Lipecka, L.G. Dubois, A. Bogeas, N. Tahiri-Jouti, H. Chneiweiss, and M.-P. Junier. Sirtuin-2 activity is required for glioma stem cell proliferation arrest but not necrosis induced by resveratrol. *Stem Cell Reviews and Reports*, 10(1):103–113, 2014b.

J.-X. Shao, T.-T. Zhang, T.-Y. Liu, Y.-Z. Quan, F. Li, J. Liu, X. Yang, Q. Xie, and W.-L. Xia. Sirtuin functions in the brain: From physiological to pathological aspects. *Journal of Shanghai Jiaotong University (Science)*, 19(6):651–662, 2014.

R.M. Starke, D.M.S. Raper, D. Ding, N. Chalouhi, G.K. Owens, D.M. Hasan, R. Medel, and A.S. Dumont. Tumor necrosis factor-α modulates cerebral aneurysm formation and rupture. *Translational Stroke Research*, 5(2):269–277, 2014.

I. Taes, M. Timmers, N. Hersmus, A. Bento-Abreu, L. Van Den Bosch, P. Van Damme, J. Auwerx, and W. Robberecht. *Hdac6* deletion delays disease progression in the $SOD1^{G39A}$ mouse model of ALS. *Human Molecular Genetics*, 22(9):1783–1790, 2013.

M. Van Meter, V. Gorbunova, and A. Seluanov. SIRT6: A promising target for cancer prevention and therapy. *Advances in Experimental Medicine and Biology*, 818:181–196, 2014.

M. Vieira, J. Fernandes, L. Carreto, B. Anuncibay-Soto, M. Santos, J. Han, A. Fernández-López, C.B. Duarte, A.L. Carvalho, and A.E. Santos. Ischemic insults induce necroptotic cell death in hippocampal neurons through the up-regulation of endogenous RIP3. *Neurobiology of Disease*, 68:26–36, 2014.

I.A. Viringipurampeer, X. Shan, K. Gregory-Evans, J.P. Zhang, Z. Mohammadi, and C.Y. Gregory-Evans. *Rip3* knockdown rescues photoreceptor cell death in blind *pde6c* zebrafish. *Cell Death and Differentiation*, 21(5):665–675, 2014.

E.B. Vitner, R. Salomon, T. Farfel-Becker, A. Meshcheriakova, M. Ali, A.D. Klein, F.M. Platt, T.M. Cox, and A.H. Futerman. RIPK3 as a potential therapeutic target for Gaucher's disease. *Nature Medicine*, 20(2): 204–208, 2014.

G. Wagner and M.D. Hirschey. Nonenzymatic protein acylation as a carbon stress regulated by sirtuin deacylases. *Molecular Cell*, 54(1):5–16, 2014.

B.R. Webster, I. Scott, J. Traba, K. Han, and M.N. Sack. Regulation of autophagy and mitophagy by nutrient availability and acetylation. *Biochimica et Biophysica Acta - Molecular and Cell Biology of Lipids*, 1841(4):525–534, 2014.

J. Wu, Z. Huang, J. Ren, Z. Zhang, P. He, Y. Li, J. Ma, W. Chen, Y. Zhang, X. Zhou, Z. Yang, S.-Q. Wu, L. Chen, and J. Han. *Mlkl* knockout mice demonstrate the indispensable role of Mlkl in necroptosis. *Cell Research*, 23(8):994–1006, 2013.

L. Wu, A. Gomes, and D. Sinclair. Geroncogenesis: Metabolic changes during aging as a driver of tumorigenesis. *Cancer Cell*, 25(1):12–19, 2014.

K. Yamagata, Y. Goto, H. Nishimasu, J. Morimoto, R. Ishitani, N. Dohmae, N. Takeda, R. Nagai, I. Komuro, H. Suga, and O. Nureki. Structural basis for potent inhibition of SIRT2 deacetylase by a macrocyclic peptide inducing dynamic structural change. *Structure*, 22(2):345–352, 2014.

X. Yan, A. Anzai, Y. Katsumata, T. Matsuhashi, K. Ito, J. Endo, T. Yamamoto, A. Takeshima, K. Shinmura, W. Shen, K. Fukuda, and M. Sano. Temporal dynamics of cardiac immune cell accumulation following acute myocardial infarction. *Journal of Molecular and Cellular Cardiology*, 62:24–35, 2013.

D.Y. Yoo, D.W. Kim, M.J. Kim, J.H. Choi, H.Y. Jung, S.M. Nam, J.W. Kim, Y.S. Yoon, S.Y. Choi, and I.K. Hwang. Sodium butyrate, a histone deacetylase inhibitor, ameliorates SIRT2-induced memory impairment, reduction of cell proliferation, and neuroblast differentiation in the dentate gyrus. *Neurological Research*, 37(1):69–76, 2015.

X. Yu, Q. Deng, A.M. Bode, Z. Dong, and Y. Cao. The role of necroptosis, an alternative form of cell death, in cancer therapy. *Expert Review of Anticancer Therapy*, 13(7):883–893, 2013.

H. Yuan, L. Su, and W. Chen. The emerging and diverse roles of sirtuins in cancer: A clinical perspective. *OncoTargets and Therapy*, 6:1399–1416, 2013.

W. Zhou and J. Yuan. Cell biology: Death by deacetylation. *Nature*, 492(7428):194–195, 2012.
