# Supplementary figures and images for: Propagation of errors in citation networks: a study involving the entire citation network of a widely cited paper published in, and later retracted from, the journal Nature
Source: Res Integr Peer Rev. 2016 May 3;1:3. doi: 10.1186/s41073-016-0008-5 (PMC5793988; doi:10.1186/s41073-016-0008-5)

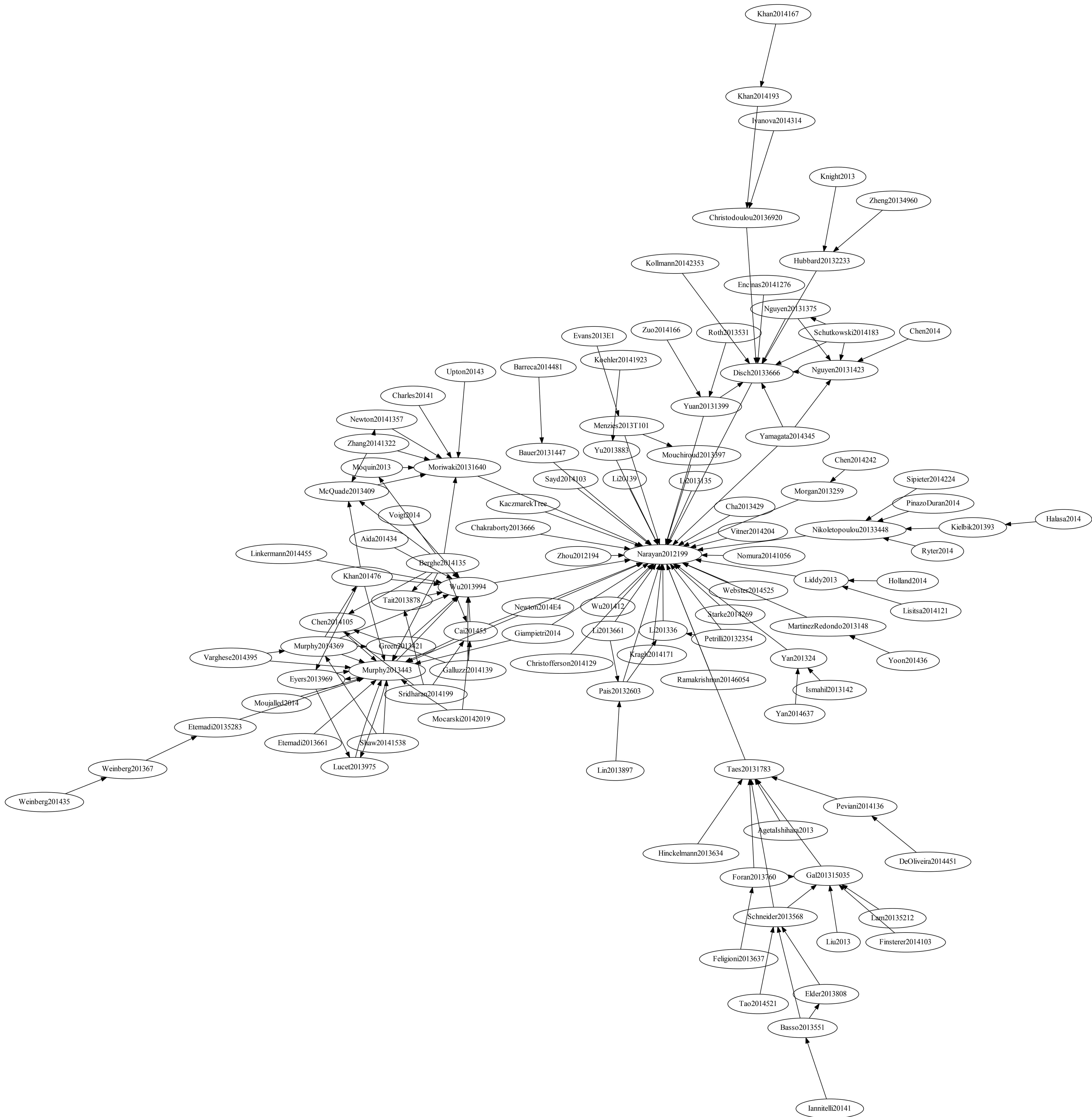

Supplement: Supplementary file 4 — The 2014 citation network with named nodes. PDF file showing the graph that constitutes the 2014 citation network with nodes labelled with their BibTE X identifiers (expanded in Additional file 6). In order to keep the figure readable, a large subgraph has been collapsed into the node “KaczmarekTree”. The latter node is expanded in Additional file 5. The figure is scalable and can be enlarged in any PDF viewer capable of zooming without becoming fuzzy. (PDF 62.1 kb) [file 41073_2016_8_MOESM4_ESM.pdf]

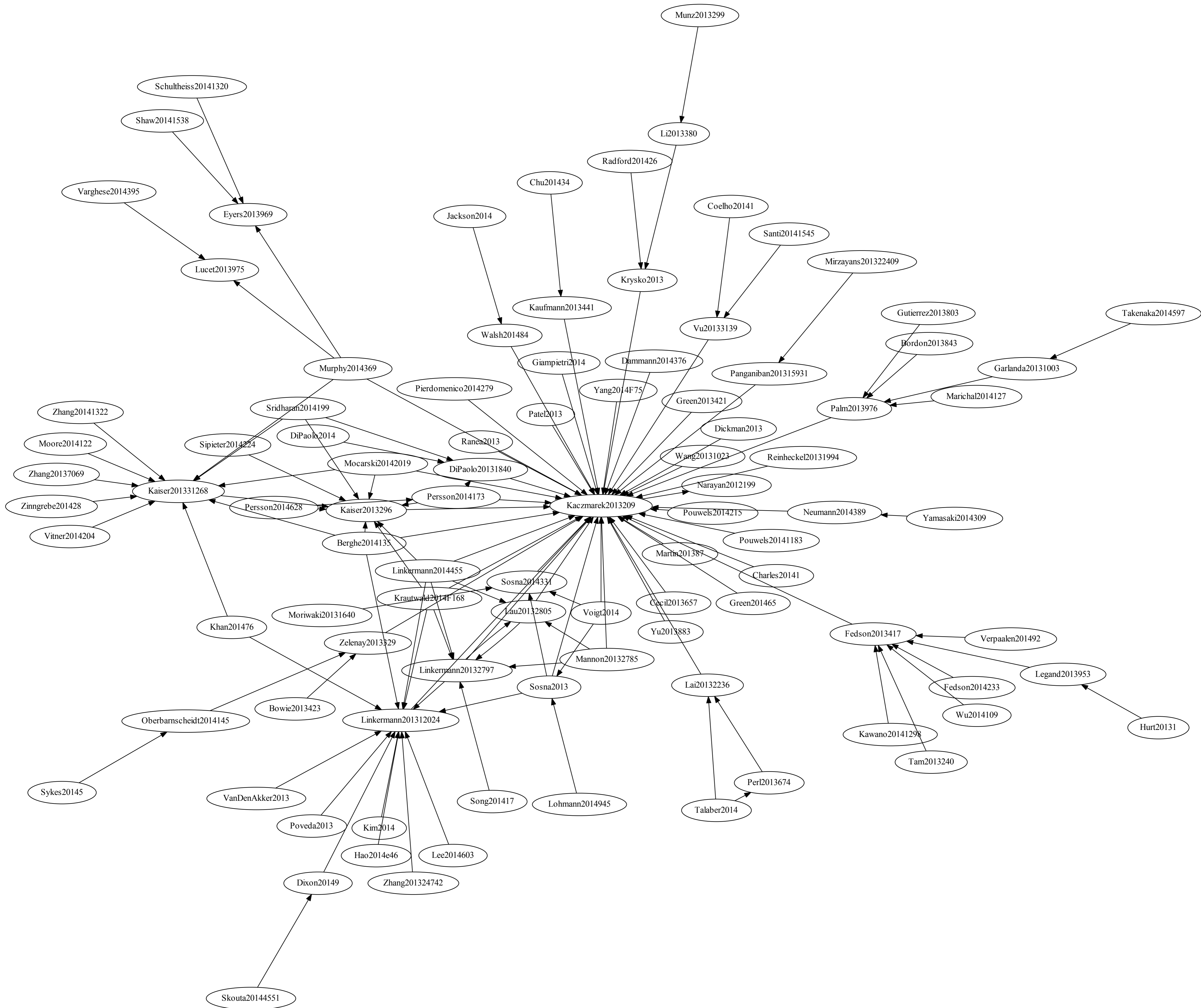

Supplement: Supplementary file 5 — Subgraph of the 2014 citation network with named nodes. PDF file showing the subgraph with named nodes of the 2014 citation network that was collapsed into the node “KaczmarekTree” in Additional file 4. The properties of this figure are as for Additional file 4. (PDF 57.7 kb) [file 41073_2016_8_MOESM5_ESM.pdf]
